# Emotion Recognition of Online Education Learners by Convolutional Neural Networks

**DOI:** 10.1155/2022/4316812

**Published:** 2022-06-09

**Authors:** Fulan Ye

**Affiliations:** School of Big Data, Fuzhou University of International Studies and Trade, Fuzhou 350202, Fujian, China

## Abstract

At present, the facial expression recognition model in video communication has problems such as weak network generalization ability and complex model structure, which leads to a large amount of computation. Firstly, the Inception architecture is adopted as a design philosophy. The Visual Geometry Group Network (VGGNet) model is improved. Multiscale kernel convolutional layers are constructed to obtain more expressive features. Secondly, the attention mechanism is integrated into a multiscale feature fusion network to form a multiattention mechanism Convolutional Neural Network (CNN) model. Novel spatial and multichannel attention models are designed. The effects of redundant information and noise are reduced. Finally, experiments are carried out on the Fer2013 dataset and the Extended Cohn-Kanade Dataset (CK+) to verify the detection accuracy of the model. The results show that the Delivered Duty Unpaid (DDU) loss can be used for facial expression recognition in complex environments. After the attention module is added, the overall recognition accuracy of the network on Fer2013 and CK+ has been improved to varying degrees. The addition of the channel attention module has a more obvious effect on the recognition accuracy compared with the spatial attention module. The addition of the attention module enables the network to increase the attention to error-prone samples. The improved network model can better extract the key features of facial expressions, enhance the feature discrimination ability, and improve the recognition accuracy of error-prone expressions. The accuracy rate of facial expression recognition with larger movements is over 98%. Facial expressions are an important way of communication between people, and online video has greatly limited this communication method. The proposed CNN model based on multiscale feature fusion will effectively solve these network limitations and have an important and positive impact on future network information exchange.

## 1. Introduction

This study aims to improve the accuracy of facial recognition. Facial expressions can intuitively convey people's emotions and wish through nontext forms and are the main method of conveying emotional information and communicating interpersonal relationships between the two parties [1]. Psychologists have shown through investigation and research that, in the process of human emotion communication, the emotional information conveyed by facial expressions accounts for about 55%, the sound form accounts for 38%, and the language form conveys only 7% of the information [2]. Facial expressions can better reflect the authenticity of human inner emotional activities [3]. Expression is an irreplaceable nonverbal communication method in interpersonal communication, which plays a role in conveying emotional state and intention [4].

At present, due to the influence of Coronavirus Disease 2019, the combination of online and offline classes has become a new development trend in teaching. The recognition of students' facial expressions can become an important technical means to assist teachers in classroom teaching and can also improve the quality of teaching. Education and teaching will also flourish towards meaningful and valuable health education [5]. The improved existing deep learning model structure is used as the entry point through the research and summary of the existing deep learning theory and network model. In view of the current network, with generalization ability being weak, the model structure is complex and causes a large amount of calculation and other problems, and facial expression recognition problems have been studied [6].

Convolutional Neural Networks (CNNs) are widely used in the field of facial expression recognition. Vu et al. designed a multimodel system, which used four different models for video expression recognition: audio model, static expression recognition model, dynamic expression recognition model, and 3D facial feature point classification model. Sound Net is used in the audio model to extract audio features from the video. In the static expression recognition model, 1 InceptionNet and 3 Dense Nets are used to extract expression features from a single video frame. In the dynamic expression recognition model, the Visual Geometry Group (VGG) network combined with the Long Short-Term Memory (LSTM) network is used to extract the temporal features of the video. In the 3D model of face feature point classification, the extracted face feature points are calculated by Euclidean distance as a feature for classification [7]. Yang and Zhang have designed a Frame Focusing Attention Network (FAN) for video facial expression recognition, identifying some discriminative frames, and highlighting them in an end-to-end framework. The network is divided into feature embedding and frame attention modules. The feature embedding module is a deep CNN. It embeds face images into feature vectors. The frame attention module learns multiple attention weights. These weights are used to adaptively aggregate feature vectors to form a single discriminative video representation [8]. Hariri has proposed a dual-modal fusion model. The model is divided into two parts: the face image and the audio model. In the face image model, four different CNNs are used for expression feature extraction, and the extracted features are input into bidirectional LSTM for temporal feature extraction [9]. In the audio model, two different methods are used to extract supplementary information from the audio. Finally, the fusion stage uses a grid search strategy to optimize the model's performance. These listed works all have some problems, including more models, which consume a lot of time and resources for training and recognition, and the recognition rate is also low.

**Figure 1:**
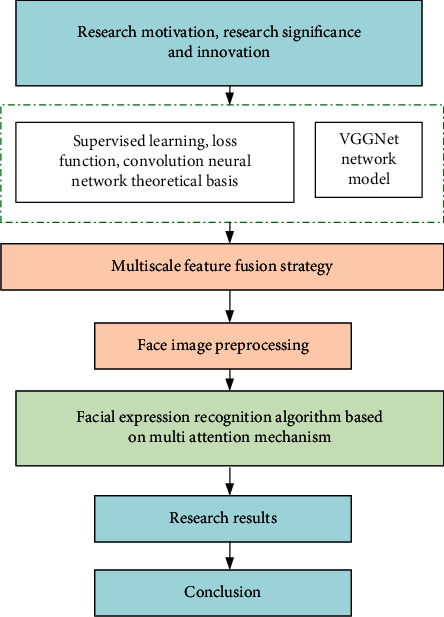
Framework of research.

Based on improving the facial recognition accuracy of current convolutional networks, this study presents a convolutional computational neural network model for the fusion of multiple large-scale information. First, the network structure is optimized and improved. The feature maps output by different convolutional layers are fused at multiple scales. Second, the loss function is improved. More efficient regularization strategies are introduced. More discriminative expression features are extracted. Aiming at the problems of randomness, noise, and insufficient discriminative features of facial expressions in the feature extraction process of CNN, a multiattention mechanism facial expression recognition algorithm is proposed. By adding a variety of improved attention modules to the network, the adaptive extraction of key feature information related to the expression recognition task is further enhanced to enhance the discriminability of expression features further, and improvements and innovations are made on top of this. The innovation is that, considering that the face information intercepted in the video is easily affected by the external environment, the attention module is introduced, and the calculation amount is smaller than the existing model. A good recognition accuracy rate is achieved. These innovations open the way for follow-up research. The overall architecture is shown in Figure 1.

## 2. Materials and Methods

### 2.1. Supervised Learning

Usually, machine learning is used to deal with computer vision optimization problems and can use the mapping function *f*. Its relevant parameter is *θ*, as shown in the following equation:

(1) $$\begin{array}{c} f_{\theta }:X⟶Y. \end{array}$$

In (1), *X* is the inlet gap, and Y is the outlet gap. As a visual recognition task in what is essentially a multiclass graph partitioning problem, the entry space is a set of two-dimensional pixels. An exit space is a set of labels or targets. Problem object-specific fixed-type ensembles are dispatched to all entries [10]. Morphologically, the goal of a supervised learning system is to approximate the mapping function *f*. A prediction model is established based on the training dataset, and all inputs are related to a certain token [11]. Assuming that there are *n* samples, the corresponding training dataset is shown as follows:

(2) $$\begin{array}{c} \left\{\left(x_{i},y_{i}\right),\ldots ,\left(x_{n},y_{n}\right)\right\}. \end{array}$$

In (2), (*x*_*i*_, *y*_*i*_) ∈ *X* × *Y*, *i* ∈ {1,…, *n*}, and the target of supervised learning is in every sample of the training dataset. In the space of function *f*, make the loss function $R\left(\hat{y_{i}},y_{i}\right)$ be the smallest *f*^*∗*^ are looking for, as shown in the following equation:

(3) $$\begin{array}{c} f^{*}\approx \underset{f\in F}{\underset{︸}{\arg\min}}\frac{1}{n}\sum_{i=1}^{n}R\left(f\left(x_{i},\theta \right),y_{i}\right). \end{array}$$

**Figure 2:**
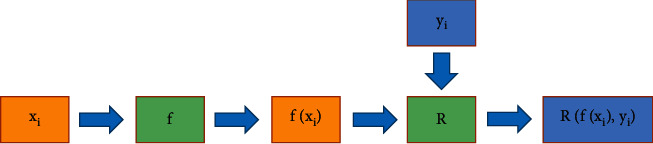
The flow of classical supervised learning.

The loss function *R* measures that the predicted labels $\hat{y_{i}}=f\left(x_{i},\theta \right)$ are inconsistent with the true labels *y*_*i*_. A typical supervised learning process is shown in Figure 2.

The performance of the model is generally reflected in the accuracy rate. The accuracy rate is the percentage of reasonably divided data samples in all data analysis samples [12]. Here, however, the focus is on the performance of the predictive model on previously observed training data or its ability to generalize on the test dataset [13].

### 2.2. Loss Function

In the classic multiclass image classification problem, the mapping function *f*_*θ*_ : *X*⟶*Y* is generally used. In each class, the input space is reflected as a likelihood distribution $\hat{y_{i}}\in Y$. The probability distribution also represents the actual labels of the dataset *y*_*i*_. For the predicted labels $\hat{y_{i}}$ of any sample in the given dataset and the corresponding actual labels *y*_*i*_, the parameter *θ* is optimized by the objective function such that the two probability distributions $\hat{y_{i}}$ are similar to *y*_*i*_ [14].

The method of measuring the dissimilarity between two probability distributions is called the Kullback-Leibler (KL) divergence [15]. KL divergence, also known as relative entropy, is shown as follows:

(4) $$\begin{array}{c} KL\left(y_{i}\|\hat{y_{i}}\right)=\sum_{k=1}^{k}y_{ik}\log\frac{y_{ik}}{\hat{y_{ik}}}. \end{array}$$

In the above equation, *y*_*ik*_=1 if *x*_*i*_ belongs to the *k*-th class and 0 otherwise. $\hat{y_{ik}}$ represents the predicted probability of the input sample entering the *k*-th class. *K* is the number of classes. It can also be rewritten, as shown in the following equation:

(5) $$\begin{array}{c} KL\left(y_{i}\|\hat{y_{i}}\right)=\sum_{k}y_{ik}\log\;y_{ik}-\sum_{k}y_{ik}\log\hat{y_{ik}}=-H\left(y_{i}\right)+H\left(y_{i},\hat{y_{i}}\right). \end{array}$$

In (5), $H\left(y_{i},\hat{y_{i}}\right)$ is called cross-entropy or negative log-likelihood. The dissimilarity between the predicted label and the true label is measured as

(6) $$\begin{array}{c} H\left(y_{i},\hat{y_{i}}\right)=-\sum_{k}y_{ik}\log\hat{y_{ik}}. \end{array}$$

In a supervised learning paradigm, minimize $H\left(y_{i},\hat{y_{i}}\right)$ using real labels and model *f*^*∗*^ with no similarity between the predictions.

### 2.3. CNN

**Figure 3:**
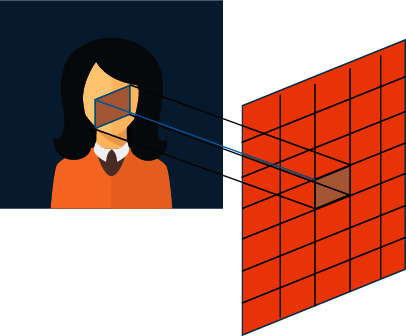
Convolution operation on the input image.

The core technology of CNN is convolution filtering. It performs local feature convolution with the input information, and the obtained feedback also manifests as local features [16]. An example of a small region of the input image being convolved is shown in Figure 3.

In the *i*th filter *f*^*∗*^, *i* ∈ {1,  2,…, *N*_*c*_}, and the response parameter is *θ*_*f*_*i*__ which is convolved with the image patch pixel value *X*. The response is saved as the filter response *f*(*θ*_*f*_*i*__, *X*). Likewise, the filter is moved along the local extent of the input image to create a 2D filtered output. Different filters are also applied to the input image to build a convolutional layer with *N*_*c*_ channels. The convolution output is passed through an activation function such as the rectified linear unit (ReLU) to extract the hidden nonlinear feature data [17].

The advantages of convolution operations in CNNs are twofold: parameter sharing and connection sparsity. Convolutional filters or feature detectors are applied to different regions of the image; that is, all parts of the image share the parameters of the *i*th filter. Furthermore, the output value of the filter only depends on a small number of input values. This situation results in sparse connections between input and output. Therefore, the designed CNN contains fewer parameters than the equivalent Deep Neural Network (DNN). Multiple convolutional layers are stacked sequentially, and a deep CNN is constructed. At the heart of this network is a deep CNN hierarchical manager that automatically learns complex image features, resulting in deep features as high-level representations that encode the abstract semantics of the data. Then, a trainable linear unit (fully connected layer) classifies the resulting deep feature vectors using a specific loss function. A large corpus of labeled data is used to learn powerful visual features to enable deep CNNs to abstract well on real data. Convolutional networks learn facial features at many different levels of abstraction, from small edges to very complex features such as nose, eye, and mouth features. The deep features are mixed with fully connected linear units of the last convolutional layer. The loss function then classifies the resulting deep features and makes predictions based on a fixed set of classes.

### 2.4. Introduction to VGGNet Model

**Figure 4:**
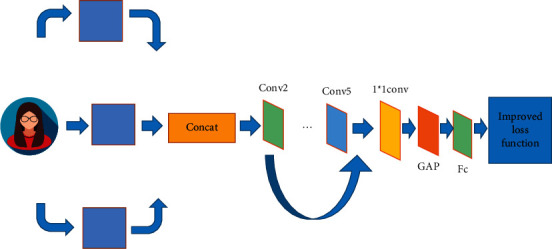
Network structure of multiscale feature fusion.

Visual Geometry Group Network (VGGNet) contains several different levels of network models. VGGNet16 is chosen as the backbone network and improved [18]. VGGNet16 is structurally improved to make the network more adaptable to the actual needs of facial expression recognition tasks [19]. The overall network structure is shown in Figure 4.

In Figure 4, in the improved model, multiscale feature fusion at different levels of network width and depth is successfully established without affecting the depth of the network [20]. In VGGNet16, since the parameters are mainly gathered on several fully connected layers at the end of the original network, all connected layers in the original network are replaced by the global average pooling method layer. A direct connection is formed between the type of the expression label in the dataset and the output type by setting a fully connected layer with a node of 7. Finally, the design results enter the Softmax classifier to obtain the best probability of each type and output the results [21].

In CNN, the number of parameters in the network is usually used as the evaluation index of the complexity of the network. The calculation of the parameter quantity in the network is shown as follows:

(7) $$\begin{array}{c} S=K^{2}\times I\times O. \end{array}$$

In the above equation, *K* is the size of the convolution kernel, and *I* and *O* are the numbers of input and output channels of the feature map, respectively.

### 2.5. Multiscale Feature Fusion Strategy

The feature maps of Cony2 and Cony3 before the input pooling layer in the convolution module are extracted. They are used as a branch feature map and VGGNet16 together with the feature map provided by the last layer of convolution module Cony5 for more scale features. Finally, the fused feature maps are integrated, and path dimensionality reduction is performed through a 1 × 1 convolutional layer [22].

In this experiment, the stochastic gradient descent algorithm updates the network parameters. The momentum is set to 0.9, the batch scale is set to 32, and the initial learning rate is set to 0.01. Subsequent experiments use an exponential decay strategy to adjust the learning rate dynamically. The learning rate decay coefficient is set to 0.9.

### 2.6. Experimental Dataset

#### 2.6.1. Fer2013 Dataset

The Fer2013 dataset is a public facial expression dataset provided by the 2013 Kaggle Facial Expression Recognition Challenge. The Fer2013 dataset contains different states of people. The face recognition accuracy on this dataset is 65 ± 5%. It is challenging to use this dataset for facial expression recognition.

#### 2.6.2. CK+ Dataset

The Extended Cohn-Kanade Dataset (CK+) was collected and proposed by Lucey in 2010 and extended based on Cohn-Kanade Dataset (CK). It is the most widely used facial expression dataset captured under controlled laboratory conditions at present [23]. In order to be compatible with the seven basic expressions in the Fer2013 dataset, contempt expressions with a small sample size were removed in this experiment, and 3 to 5 frames were intercepted from each image sequence as expression samples in this experiment [24]. Finally, the obtained data samples are randomly divided into a training set and a test set according to the ratio of 9 : 1.

### 2.7. Face Image Preprocessing

#### 2.7.1. Face Detection

**Figure 5:**
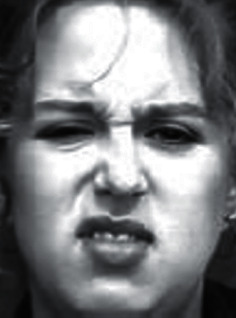
Cropped face image.

In addition to the human face, the facial expression images in the given dataset also include nonface regions such as the background [25]. Therefore, the input image is preprocessed before being fed into the CNN. The detected face regions are cropped and saved [15]. The valid images obtained after the face images in the dataset are preprocessed as shown in Figure 5.

In Figure 5, the existence of a face is determined by multiple stacking of face candidate windows of different scales to reduce the false detection rate of a face. The scale of the detected image is normalized by the bilinear interpolation algorithm. A grayscale image of 224 × 224 pixels is output and saved [26].

#### 2.7.2. Data Enhancement

**Figure 6:**
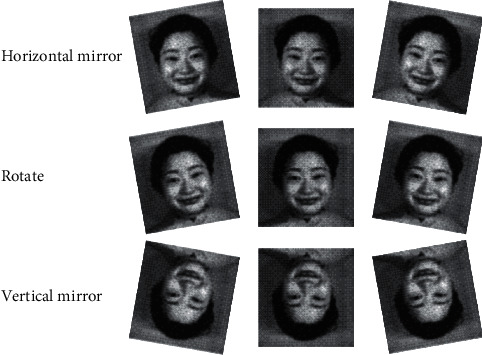
Operation of data augmentation.

When the sample size is seriously insufficient, it is necessary to artificially increase the sample size. Data augmentation is used to augment the dataset samples. Without adding additional image samples, many completely new image samples are generated without changing the sample label category [27]. The data enhancement effect is shown in Figure 6.

In Figure 6, in order to improve the anti-interference and reliability of the model, each pixel of the data is mirrored and expanded to three times the number of original samples. The resulting pixels are then angularly flipped. The rotation angle range is set to ±100. After that, flip every fifty degrees. Next, the pixels are normalized. The pixel size is set to 224 × 224, which enlarges the number of samples by 15 times.

### 2.8. Facial Expression Recognition Algorithm Based on Multiattention Mechanism

In CNN, it is generally assumed that all position information on the feature map is equally important. But this does not necessarily give great results when extracting image features. This is because the content on each face image is not the same, and different tasks focus on the image content differently, focusing on the extraction of facial expression features. Therefore, different positions of the input image are given the same weight indiscriminately. This will not only extract the facial expression feature information but also extract a lot of redundant background noise information, which will eventually affect the recognition accuracy. The attention mechanism is introduced into the network model so that the network suppresses redundant information and enhances important information when extracting features, making the expression recognition results more accurate.

The attention mechanism originated from the study of the human visual system. Human vision quickly scans global information to obtain target areas that need to be focused on. This area is also known as the focus of attention. Then, more attention resources are allocated to this area to obtain more detailed information about the relevant target. The above mechanism is often referred to as the attention mechanism, which is a signal processing mechanism unique to human vision. In the case of limited information processing resources, this mechanism can selectively focus on specific parts within the visual range, capture the most discriminative visual information, and improve the efficiency and accuracy of information processing.

Inspired by the human visual attention mechanism, many researchers have begun to try to introduce the attention mechanism into the neural network so that the computer can also strengthen the attention to the key information like humans. At present, the attention mechanism has been widely used in various types of deep learning tasks, such as natural language processing, image recognition, and speech recognition. In CNN, attention modules are usually generic and can be embedded into existing network architectures to obtain more discriminative features by assigning different weights to different regions of the feature map. According to the different forms of attention, attention is divided into soft attention and hard attention. After generating the attention weights, hard attention will set a part of the unqualified weights to 0 and no longer pay attention to this part of redundant information that is not related to the current task. Soft attention avoids filtering data and calculates attention weights on all data.

### 2.9. Channel Attention Mechanism

The input image is initially represented by three channels: Red (R), Green (G), and Blue (B). Each convolution kernel extracts different features from the input image and extracts a set of feature maps with the number of channels equal to the number of convolution kernels. The features of each channel represent the components of the image on different convolution kernels. The features of different channels have different degrees of influence on key information. The channel attention mechanism is used to automatically acquire channel features that are more critical to the current task.

**Figure 7:**
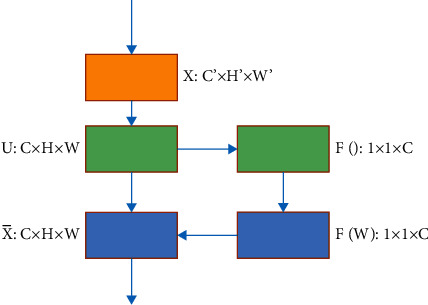
Structure of SENet model.

The Squeeze-and-Excitation Networks (SENet) model mainly focuses on the feature channel perspective. The importance of each feature channel can be obtained, and then the learned features can be weighed by processing by using the interdependence between the explicit model feature channels. The SE module mainly includes two operations: squeeze and excitation. The SENet model structure is shown in Figure 7.

In Figure 7, convolution can only be applied to local space, so it is difficult to obtain sufficient information to extract the correlation between channels. Therefore, in the SE module, all input images are preprocessed by a pooling layer of global spatial average, and the representation of each channel is based on the global spatial characteristics of each channel to establish a one-dimensional channel descriptor that contains the global receptive field information of each channel to some extent. Assuming that the input feature map is *U*=*R*^*H*×*W*×*C*^, the channel descriptor *z* ∈ *R*^1×1×*C*^ is obtained after the pooling layer. Then, the *c*-th output of *z* is shown as follows:

(8) $$\begin{array}{c} z_{c}=\frac{1}{H\times W}\sum_{i=1}^{H}\sum_{j=1}^{W}u_{c}\left(i,j\right). \end{array}$$

In the above equation, *u*_*c*_ is the output of the *c*-th convolution kernel after the feature map *U* undergoes a standard convolution. After compression, the global description features of each channel are obtained. Then, the dependencies between different channels are obtained through the activation operation. A network layer with parameter *w* is used to generate weights for all feature channels. The specific process is as follows: Firstly, the compressed output will go through a fully connected layer with *c*/*r* nodes. *r* is a scaling parameter used to reduce the number of channels and the amount of computation. The ReLU function is used to add nonlinear transformations. Then, a fully connected layer with the number of nodes *c* is used to restore the original dimension. Finally, after the sigmoid function is activated, the learned weights of each channel are obtained. In order to output the feature map adjusted by the SE module, the weight value output by the activation process is regarded as the importance of each feature channel. The weights of each channel are multiplied by the previous features to recalibrate the input features. The SE module is lightweight and consists of two fully connected layers and a global average pooling layer, which increases the sensitivity of the model to channel features while only increasing the number of parameters and computation, resulting in significant performance improvements.

### 2.10. Spatial Attention Mechanism

**Figure 8:**
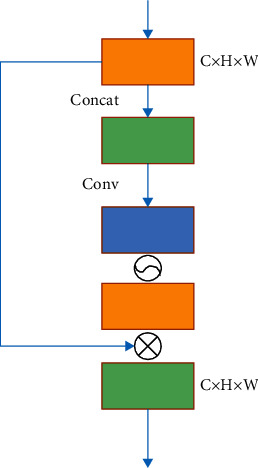
Structure of spatial attention module.

In order to find the spatial structure attention, the importance of each position in the graph is learned at the spatial structure level. The feature map generated by the first channel attention modeling is used as the entry feature map and the global mean pooling method according to the channel level and operation of the global max pooling method. Afterwards, these two feature maps are combined in series. A standard convolutional layer with a kernel length of 7 × 7 is used to perform dimensionality reduction of the spatial structure channel. Sigmoid function activations are used to form a two-dimensional attention map. Then, the spatial structure attention map and the feature map of the entrance are calculated by the Hadamard product to obtain the feature map of the final output. The structure of the spatial attention module in the model is shown in Figure 8.

Assume that the input feature map is *F*. Then, the calculation of the spatial attention map is shown as follows:

(9) $$\begin{array}{c} M_{s}\left(F\right)=\sigma \left(f^{7\times 7}\left(\left[F_{\text{avg}}^{S};F_{\text{max}}^{S}\right]\right)\right), \end{array}$$

where *f*^7×7^ indicates the convolution operation, the size of the convolution kernel is 7 × 7, *σ* indicates the sigmoid activation function, and the dimensions of the intermediate feature maps are all *H* × *W* × 1.

## 3. Results and Discussion

### 3.1. Comparison of Loss Functions

**Figure 9:**
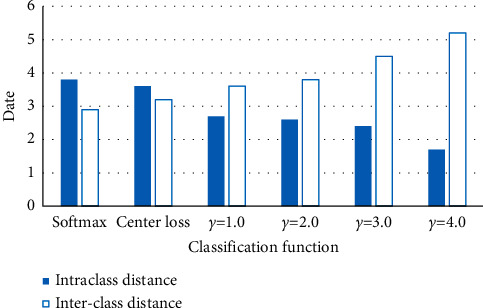
Differences in intra- and interclass distances under different loss functions.

On a mixed dataset consisting of CK+ and Fer2013, more extensive experiments are carried out to evaluate the properties of the provided loss functions. The Delivered Duty Unpaid (DDU) loss function is introduced. It has better properties compared to baseline loss functions (i.e., Softmax economic loss and center economic loss). Secondly, the resulting DDU loss is evaluated in the hybrid dataset and in the two large Fer datasets in a variety of better ways.  Figure 9 shows the difference between intra- and interclass distances under various loss functions; the smaller the intraclass distance deviation, the larger the interclass distance and the better the performance of the loss function.

In Figure 9, due to the increase of the *γ*-like value, the contribution of the DDU loss also increases accordingly, and the spacings embedded in the voids are also larger and larger. Feature groups are usually compact and well segmented. When using the hyperparameter *γ*, feature clusters are biased away from the set of other features. Therefore, DDU loss can be used for facial expression recognition in complex environments. The DDU loss implicitly pushes the deep features of a class from other classes to the corresponding class centers in the embedding space. Under the joint supervision of Softmax loss and center loss, DDU loss has extremely uneven data distribution in the embedding space. This effectively distinguishes feature clusters from the majority and minority classes.

### 3.2. Validation of Attention Mechanism

**Figure 10:**
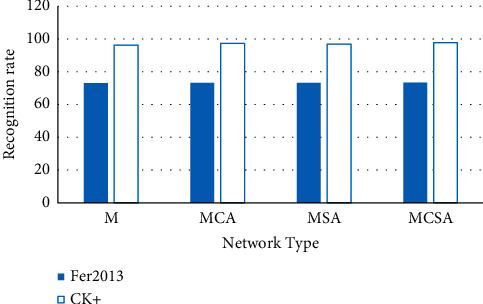
Comparison of recognition rates of various attention networks on different datasets.

The comparative experiments are carried out by adding different categories of attention modules to the multiscale feature fusion network model proposed above, which are as follows: (1) M-VGGNet, a multiscale feature fusion network model that does not introduce an attention mechanism; (2) MCA-VGGNet, where only the network model of channel attention (CA) mechanism is introduced; (3) MSA-VGGNet, a network model that only introduces the spatial attention (SA) mechanism; and (4) MCSA-VGGNet, where the spatial channel attention (CSA) mechanism is introduced into the network model. The comparative experimental results are shown in Figure 10.

In Figure 10, after adding the attention module, the overall recognition accuracy of the network on the Fer2013 and CK+ datasets has been improved to varying degrees. Among them, the effect of adding a channel attention module to the recognition accuracy is more obvious than that of the spatial attention module. The experimental results showed that the superimposed use of two attention modules in MCSA-VGGNet can significantly improve recognition accuracy. The network extracts the feature information more relevant to the facial expression recognition task under the combined action of the channel and space dimensions so that the obtained expression features have stronger discriminability. The experimental results demonstrate the effectiveness of the joint use of the two attention modules.

In order to test the enhancement effect of the model on the recognition rate of various expressions after introducing the attention mechanism, three more obvious expressions are selected. Angry, happy, and sad are used as contrasting expressions. The comparison results on the two datasets are shown in Table 1.

**Figure 11:**
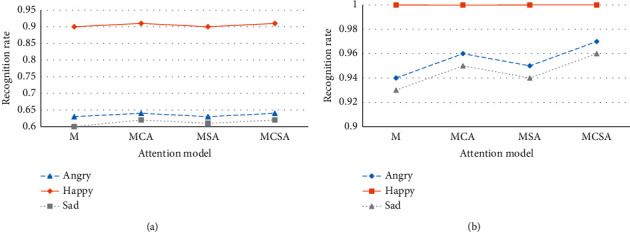
Comparison of the accuracy of different attention models for various types of expression recognition on different datasets. (a) Comparison of the accuracy of different attention models on the Fer2013 dataset for various types of expression recognition. (b) Comparison of the accuracy of different attention models on the recognition of various expressions on the CK+ dataset.

According to Table 1, the comparison of the recognition accuracy of various expressions on the two datasets by the model with different attention modules is drawn, as shown in Figure 11.

In Figure 11, after two attention modules are added to the network, the recognition accuracy of various expressions on the two datasets has been improved to a certain extent. This shows that the addition of the attention module enables the network to increase the attention to error-prone samples and extract more discriminative expression features, thereby improving the overall recognition accuracy of the model. However, expressions such as happy and angry, which have already achieved a high recognition rate, have no obvious improvement effect. This is mainly because such expression features are highly recognizable, and the features are easy to be extracted, even if the attention mechanism is not introduced. The network can still extract enough discriminative features to classify such expressions correctly.

**Figure 12:**
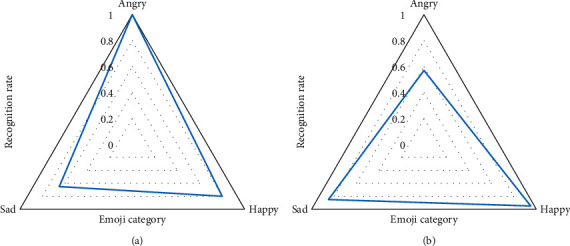
Recognition results of expressions on different datasets. (a) Recognition results on the Fer2013 model. (b) Recognition results on the CK+ model.

The validation set of the Fer2013 model consists of 50 images obtained from the network. Each image varies in size and clarity. The expressions in the validation set cover the seven basic expression types in the dataset. 30 of them are randomly selected as examples. The most obvious facial expressions are angry, sad, and happy, with ten pictures for each emotion. These face images are input into the model trained by the Fer2013 training set for expression recognition, and the corresponding expression recognition results are obtained, respectively. The data in Figure 12(a) are the average number of 30 image recognitions. The CK+ validation set consists of 50 face expression images randomly intercepted from the dataset that did not participate in the training process. The selected face image also contains seven basic expression types, and the recognition results on this dataset are shown in Figure 12(b).

In Figure 12, the recognition rate of expressions with relatively exaggerated and large movements is quite high, and the accuracy rate can even reach more than 98%. The recognition rate of facial expressions that are not suitable for showing traces is not ideal, so the later technical research direction should be closer to such expressions.

## 4. Conclusions

Due to the rapid development of computers, people have put forward higher demands on the intelligence level of HCI. Realizing the correct recognition of facial expressions by computers in HCI has become a current research trend. This study provides a facial expression recognition algorithm based on multiscale feature fusion technology, constructs a feature fusion network in dimensions, and provides a facial expression recognition algorithm based on a multiattention mechanism. Experiments are performed on the Fer2013 and CK+ datasets. The experimental results show that the algorithm effectively improves the accuracy of model recognition. Although this study has achieved certain results, there are still shortcomings. There is still a certain gap between the used dataset and the real scene, and the dataset should be closer to the real scene. Although the network structure of VGGNet has been improved to a certain extent, there are still many parameters in the improved network. The structure of the added attention module needs to be improved, and the ways of adding various attention mechanisms need to be explored. Additionally, fewer datasets are used. In the future, the latest datasets will be added, such as AffectNet, Ascertain, and Emoti. Network parameters will also be increased to explore more ways to add attention mechanisms, adding more datasets for model performance testing. This study expects optimizing the expression recognition technology further. Follow-up research will also be combined with the current development of Coronavirus Disease 2019, adding research on facial mask recognition.

## Figures and Tables

**Table 1:** Performance comparison of different models.

Algorithm type	M	MCA	MSA	MCSA
Angry (Fer2013)	0.63	0.64	0.63	0.64
Happy (Fer2013)	0.9	0.91	0.9	0.91
Sad (Fer2013)	0.6	0.63	0.62	0.63
Angry (CK+)	0.94	0.96	0.95	0.97
Happy (CK+)	1	1	1	1
Sad (CK+)	0.93	0.95	0.94	0.96

## Data Availability

The data used to support this study are available from the corresponding author upon request.

## References

[1] Tahir F. S., Abdulrahman A. A., Hikmet Thanon Z. (2022). Novel face detection algorithm with a mask on neural network training. *International Journal of Nonlinear Analysis and Applications*.

[2] Laith S., Tahir F. S., Abdulrahman A. A. (2022). Effectiveness of new algorithms for facial recognition based on deep neural networks. *International Journal of Nonlinear Analysis and Applications*.

[3] Lu C.-T., Su C.-W., Jiang H.-L., Lu Y.-Y. (2022). An interactive greeting system using convolutional neural networks for emotion recognition. *Entertainment Computing*.

[4] Armingol E., Officer A., Harismendy O., Lewis N. E. (2021). Deciphering cell-cell interactions and communication from gene expression. *Nature Reviews Genetics*.

[5] Cutri R. M., Mena J. (2020). A critical reconceptualization of faculty readiness for online teaching. *Distance Education*.

[6] Jeevan G., Zacharias G. C., Nair M. S., Rajan J. (2022). An empirical study of the impact of masks on face recognition. *Pattern Recognition*.

[7] Vu H. N., Nguyen M. H., Pham C. (2022). Masked face recognition with convolutional neural networks and local binary patterns. *Applied Intelligence*.

[8] Yang X., Zhang W. (2022). Heterogeneous face detection based on multi‐task cascaded convolutional neural network. *IET Image Processing*.

[9] Hariri W. (2022). Efficient masked face recognition method during the covid-19 pandemic. *Signal, image and video processing*.

[10] Jaiswal A., Babu A. R., Zadeh M. Z., Banerjee D., Makedon F. (2020). A survey on contrastive self-supervised learning. *Technologies*.

[11] Pfaff J., Filippov A., Liu S. (2021). Intra prediction and mode coding in VVC. *IEEE Transactions on Circuits and Systems for Video Technology*.

[12] Tuzimski T., Szubartowski S. (2021). Application of d-SPE before SPE and HPLC-FLD to analyze bisphenols in human breast milk samples. *Molecules*.

[13] Muthukumar V., Narang A., Subramanian V., Belkin M., Hsu D., Sahai A. (2021). Classification vs. regression in overparameterized regimes: does the loss function matter. *Journal of Machine Learning Research*.

[14] Sun W., Wang H., Gu Q., Rong S., Fan L. (2021). Exact frequency estimation in the i.i.d. Noise via KL divergence of accumulated power. *IEEE Communications Letters*.

[15] Lindsay G. W. (2021). Convolutional neural networks as a model of the visual system: Past, present, and future. *Journal of Cognitive Neuroscience*.

[16] Cong I., Choi S., Lukin M. D. (2021). Quantum convolutional neural networks. *Nature Physics*.

[17] Zou D., Cao Y., Zhou D., Gu Q. (2020). Gradient descent optimizes over-parameterized deep ReLU networks. *Machine Learning*.

[18] Subrahmanyeswara Rao B. (2020). Accurate leukocoria predictor based on deep VGG‐net CNN technique. *IET Image Processing*.

[19] Wang Z., Liu R. M., Huang Q. T. (2020). Inflated VGGNet-16 networks for human action recognition. *Journal of Beijing University of Chemical Technology (Natural Science Edition)*.

[20] Sun Y., Weng Y., Luo B. (2020). Gesture recognition algorithm based on multi‐scale feature fusion in RGB‐D images. *IET Image Processing*.

[21] Wang X., Zhang S., Wang S., Fu T., Shi H., Mei T. (2020). Mis-classified vector guided softmax loss for face recognition. *Proceedings of the AAAI Conference on Artificial Intelligence*.

[22] Zebari R., Abdulazeez A., Zeebaree D., Zebari D., Saeed J. (2020). A comprehensive review of dimensionality reduction techniques for feature selection and feature extraction. *Journal of Applied Science and Technology Trends*.

[23] Singh A., Prakash S., Kumar A., Kumar D. (2022). A proficient approach for face detection and recognition using machine learning and high‐performance computing. *Concurrency and Computation: Practice and Experience*.

[24] Kusuma G. P., Jonathan J., Lim A. P. (2020). Emotion recognition on fer-2013 face images using fine-tuned vgg-16. *Advances in Science, Technology and Engineering Systems Journal*.

[25] Khan M. J., Khan M. J., Siddiqui A. M., Khurshid K. (2022). An automated and efficient convolutional architecture for disguise-invariant face recognition using noise-based data augmentation and deep transfer learning. *The Visual Computer*.

[26] Triwijoyoa B. K., Adila A. (2021). Analysis of medical image resizing using bicubic interpolation algorithm. *Jurnal Ilmu Komputer*.

[27] Dias W., Andaló F., Padilha R. (2022). Cross-dataset emotion recognition from facial expressions through convolutional neural networks. *Journal of Visual Communication and Image Representation*.

